# Characterization of Sigma-2 Receptor—Specific Binding Sites Using [^3^H]DTG and [^125^I]RHM-4

**DOI:** 10.3390/ph15121564

**Published:** 2022-12-15

**Authors:** Chi-Chang Weng, Aladdin Riad, Brian P. Lieberman, Kuiying Xu, Xin Peng, John L. Mikitsh, Robert H. Mach

**Affiliations:** 1 HARC and Department of Medical Imaging and Radiological Sciences, Chang Gung University, Taoyuan 333, Taiwan; 2 Department of Nuclear Medicine and Center for Advanced Molecular Imaging and Translation, Linkou Chang Gung Memorial Hospital, Taoyuan 333, Taiwan; 3 Division of Nuclear Medicine and Clinical Molecular Imaging, Department of Radiology, Perelman School of Medicine, University of Pennsylvania, Philadelphia, PA 19104, USA

**Keywords:** Sigma-2 receptor, [^3^H]DTG, [^125^I]RHM-4, (+)-pentazocine, σ1R masking procedure

## Abstract

The sigma-2 receptor/transmembrane protein 97 (σ2R/TMRM97) is a promising biomarker of tumor proliferation and a target for cancer therapy. [^3^H]DTG has been used to evaluate σ2R/TMEM97 binding affinity in compound development studies. However, [^3^H]DTG has equal and moderate binding affinities to both sigma 1 receptor (σ1R) and σ2R/TMEM97. Furthermore, co-administration with the σ1R masking compound (+)-pentazocine may cause bias in σ2R/TMEM97 binding affinity screening experiments. We have developed a radioiodinated ligand, [^125^I]RHM-4, which has high affinity and selectivity for σ2R/TMEM97 versus σ1R. In this study, a head-to-head comparison between [^3^H]DTG and [^125^I]RHM-4 on the binding affinity and their effectiveness in σ2R/TMEM97 compound screening studies was performed. The goal of these studies was to determine if this radioiodinated ligand is a suitable replacement for [^3^H]DTG for screening new σ2R/TMEM97 compounds. Furthermore, to delineate the binding properties of [^125^I]RHM-4 to the σ2R/TMEM97, the structure of RHM-4 was split into two fragments. This resulted in the identification of two binding regions in the σ2R, the “DTG” binding site, which is responsible for binding to the σ2R/TMEM97, and the secondary binding site, which is responsible for high affinity and selectivity for the σ2R/TMEM97 versus the σ1R. The results of this study indicate that [^125^I]RHM-4 is an improved radioligand for in vitro binding studies of the σ2R/TMEM97 versus [^3^H]DTG.

## 1. Introduction

The sigma receptor was discovered over 40 years ago and was originally reported as a subtype of opiate receptors [1,2]. Subsequent studies demonstrated that the sigma receptor did not behave as a traditional opioid receptor and was further classified into two different subtypes: the sigma-1 receptor (σ1R) and the sigma-2 receptor (σ2R) [3,4,5,6,7]. σ1R was cloned from different sources, such as guinea pig liver [1], human placental choriocarcinoma cells [5], human brain [8], rat brain [9,10], and mouse brain [11], and is well characterized. On the other hand, σ2R remained uncharacterized until 2017, when it was reported to be the transmembrane protein 97 (TMEM97) [12]. This important discovery led to the renaming of this protein as the σ2R/TMEM97. Our group previously reported that the σ2R/TMEM97 is a good biomarker for measuring the proliferative status of cancer cells, which is defined as the ratio of the density of receptors in proliferating (P) versus quiescent (Q) cancer cells [13]. The σ2R receptor has also been proposed as a potential target for tumor therapy [14]. For this reason, many efforts have been made to search for new σ2R ligands as potential imaging agents or as novel tumor therapeutic ligands. More recent studies have identified the σ2R/TMEM97 as having a key role in the cellular uptake of LDL via the formation of a trimeric complex with the LDL receptor and progesterone receptor membrane component-1 (PGRMC1). Other studies have shown that σ2R/TMEM97 ligands may be useful in the treatment of a number of neurological disorders, including Alzheimer’s disease [15], Huntington’s disease [16], neuropathic pain [17], and alcohol use disorder [18,19].

The study by Alon et al. [12] confirming the identity of σ2R as TMEM97 prompted us to conduct a series of studies aimed at exploring the properties of this protein in HeLa cells. In the first study, TMEM97 in HeLa cells was knocked out using CRISPR/Cas gene editing, and the binding of [^125^I]RHM-4 and [^3^H]DTG was measured in wild-type and engineered cells. We discovered that knocking out TMEM97 completely eliminated the specific binding of [^125^I]RHM4, whereas [^3^H]DTG retained a low-affinity binding site having a Kd value of ~300 nM [20]. We next evaluated the cytotoxicity of known σ2R ligands in TMEM97-knocked out HeLa cells and discovered that there was no effect on the EC_50_ value in cytotoxicity assays. These data suggest that the therapeutic effect of σ2R/TMEM97 ligands is not related to the σ2R/TMEM97 [21]. Furthermore, our results provide a rational explanation as to why there is a large difference between the Ki value of σ2R ligands in radioligand binding assays at their respective EC_50_ values in cytotoxicity assays [21].

[^3^H]DTG has been proposed as being the “gold standard” for σ2R ligand screening [22]. However, DTG was shown to have good affinity for both σ1R (Ki = 35.5 nM) and σ2R/TMEM97 (Ki = 39.9 nM), respectively [23]. Therefore, when this radioligand is used in σ2R/TMEM97 ligand screening experiments, (+)-pentazocine must be added to mask binding to the σ1R. However, recently published data have questioned the usefulness of this masking procedure with (+)-pentazocine in the [^3^H]DTG binding studies and suggested that (+)-pentazocine may interfere with the σ2R/TMEM97 ligand screening results. Moreover, according to some published results, inconsistent amounts of (+)-pentazocine, such as 100-, 200-, or 1000 nM, can affect the measured binding affinity of DTG to σ2R/TMEM97 and possibly result in inaccurate Ki values from compound screening [24].

To address the issues described above, we conducted a series of σ2R/TMEM97 binding studies comparing the properties of [^125^I]RHM-4 and [^3^H]DTG. The results of our studies indicate that [^125^I]RHM-4 is superior to [^3^H]DTG in σ2R/TMEM97 radioligand binding assays. We also present data identifying two different binding sites in the σ2R/TMEM97, the “DTG” binding site and a secondary binding site which may be important for σ2R/TMEM97 versus σ1R selectivity.

## 2. Results

### 2.1. Saturation Binding Assay

**Figure 1:**
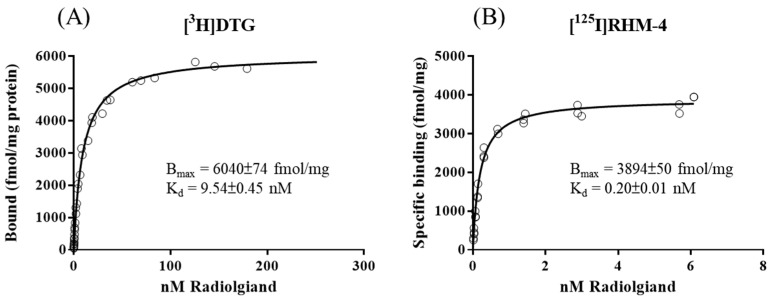
Saturation binding curves of [^3^H]DTG (**A**) and [^125^I]RHM-4 (**B**) on SD rat liver membranes. Both groups were performed with n = 3.

The saturation binding results for [^3^H]DTG and [^125^I]RHM-4 are shown in Figure 1. Both ligands performed a comparable binding capacity on SD rat liver membrane (6040 ± 74 fmol/mg for [^3^H]DTG, 3894 ± 90 fmol/mg for [^125^I]RHM-4); however, [^125^I]RHM-4 showed a much higher affinity to σ2R/TMEM97 on liver membrane homogenates compared to [^3^H]DTG (Kd = 0.2 nM for [^125^I]RHM4 vs. Kd = 9.45 nM for [^3^H]DTG).

### 2.2. Competitive Receptor Binding Assays

**Figure 2:**
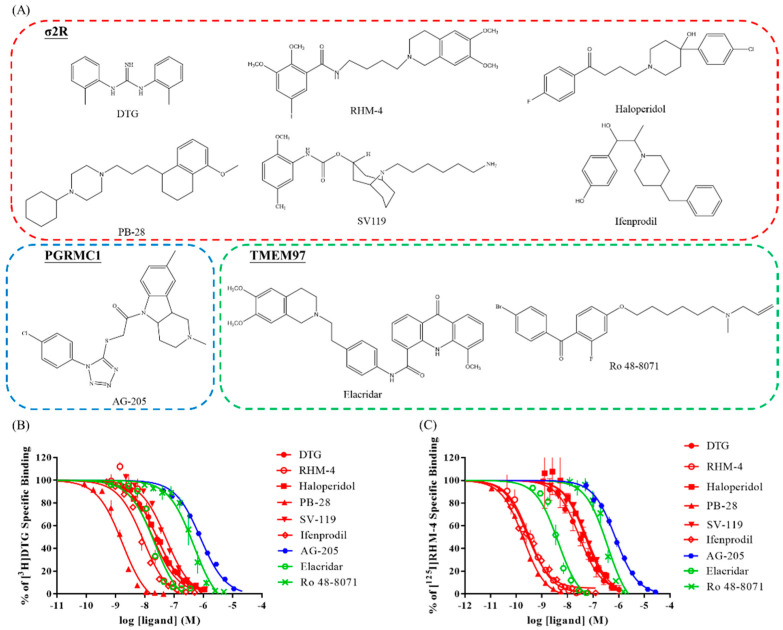
In vitro competition binding curves of different nonradioactive ligands. (**A**) The different cold ligand structures used in this assay. The competition curves with [^3^H]DTG (**B**) and [^125^I]RHM-4 (**C**).

The σ2R/TMEM97 affinity screening results for [^3^H]DTG and [^125^I]RHM-4 for the different nonradioactive ligands used in the present study are shown in Figure 2. To further characterize the binding properties of these ligands, we chose compounds that have been described in the literature as belonging to the following groups: σ2R ligands, PGRMC1, and TMEM97 inhibitors. The competition curves shown in Figure 2B,C demonstrate that the described as σ2R and TMEM97 ligands exhibited a much better inhibition affinity for [^3^H]DTG and [^125^I]RHM-4, whereas AG205 had a lower affinity for these radioligands (Ki = 807 nM for [^3^H]DTG, and 570.6 nM for [^125^I]RHM-4). These results are consistent with the two radioligands binding specifically to σ2R/TMEM97 but not to PGRMC1. These results are also consistent with our prior observations on the binding properties of [^125^I]RHM-4 and [^3^H]DTG in TMEM97 k/o, PGRMC1 k/o, and TMEM97/PGRMC1 double k/o cells [20]. The Ki values for each cold ligand are listed in Table 1.

**Figure 3:**
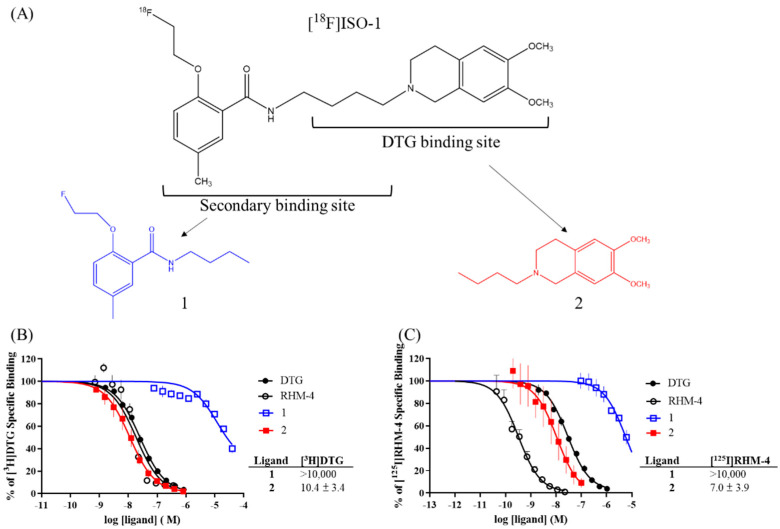
The RHM-4 derivative, [^18^F]ISO-1, was split into two different compounds (**A**); the competition binding results with [^3^H]DTG (**B**) and [^125^I]RHM-4 (**C**).

To further explore which ligand moiety may enhance σ2R/TMEM97 binding affinity, different fragments from the RHM-4 structure (**1** and **2 [25]**) were synthesized in our laboratory and applied in this study. Their affinity for σ2R/TMEM97 was measured with [^3^H]DTG and [^125^I]RHM-4. As shown in Figure 3, we found that the critical binding pocket for σ2R/TMEM97 was **2** (which we call the “DTG binding site”) and not **1**. The combination of fragments 1 and 2 to give RHM-4 increased the σ2R/TMEM97 affinity by approximately 10-fold, indicating the importance of the secondary binding site for σ2R/TMEM97 binding affinity. Since **2** binds to the “DTG binding site”, we also measured its affinity for the σ1R using [^3^H](+)-pentazocine. As expected, **2** had a Ki value of 38.0 ± 3.3 nM in displacing this radioligand to the σ1R, whereas **1** had a much lower potency in this assay (Ki = 1307 ± 87 nM).

**Figure 4:**
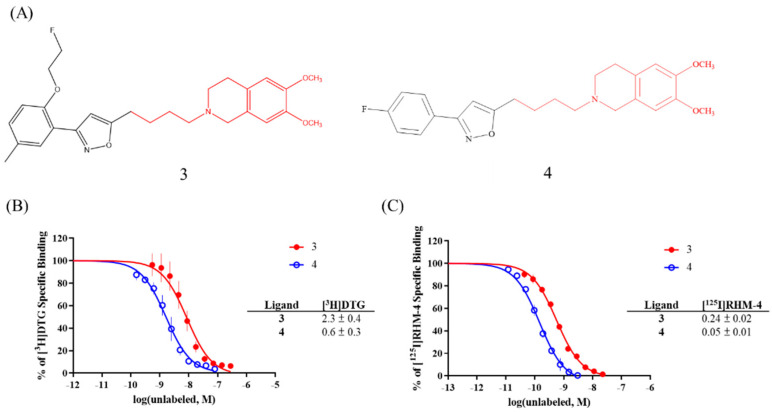
(**A**) The chemical structures of **1** and **2**; the competition binding results with (**B**) [^3^H]DTG or (**C**) [^125^I]RHM-4.

To further explore the secondary binding site, we prepared two ligands, **3** and **4**; the inhibition potency for the σ2R/TMEM97 (shown in Figure 4) was comparable to or higher than that of RHM-4, which proved that the secondary binding site played a critical role in the development of high-affinity σ2R/TMEM97 ligands. Furthermore, **3** and **4** had a relatively low affinity for σ1R (Ki = 715 ± 46 and 800 ± 23 nM, respectively), indicating the importance of the interaction of the ligands with the secondary binding site for generating compounds having a high selectivity for σ2R/TMEM97 versus σ1R.

## 3. Discussion

In the past decade, σ2R/TMEM97 has been identified as a potential target for the imaging and treatment of cancer. More recent studies have suggested that the σ2R is a potential target as a disease-modifying therapy in Alzheimer’s disease by preventing the binding of Aβ oligomers to binding sites on neurons [15,26]. Other studies have identified σ2R/TMEM97 ligands as being potentially useful in treating neuropathic pain and alcohol use disorder [17,18,19]. In the search for novel or promising σ2R/TMEM97-specific ligands, the combination of [^3^H]DTG and (+)-pentazocine, the σ1R masking reagent, has been widely used for screening. However, Abbas et al. have raised the possible disadvantage of this widely used method; the masking reagent could interfere with the dissociation constant of [^3^H]DTG as well as the new σ2R/TMEM97 compound screening results [24]. The present study compared the σ2R/TMEM97 binding affinities of [^3^H]DTG and the σ2R/TMEM97-specific radioiodinated ligand, [^125^I]RHM-4. Based on the study results, the data presented here indicate that the radioiodinated ligand is a promising σ2R/TMEM97 radioligand having ~50-fold higher σ2R/TMEM97 affinity than that of [^3^H]DTG. Moreover, because of the high σ2R/TMEM97 specificity of [^125^I]RHM-4, there is no need to mask σ1R by adding (+)-pentazocine, which makes this screening procedure more convenient, cost-effective, and time-saving.

To further verify the usefulness of σ2R/TMEM97 ligand affinity screening, a full competition assay using both radioligands, [^3^H]DTG and [^125^I]RHM-4, was performed on a panel of compounds reported as σ2R-, TMEM97-, and PGRMC1-specific compounds. As expected, the overall ligand affinity screening results were comparable between these two radioligands. All σ2R- and TMEM97-specific ligands displaced both radioligands, whereas the PGRMC1-specific ligand, AG-205, revealed a σ2R affinity close to 1μM, which is consistent with the reports that PGRMC1 and σ2R are two different proteins [27,28]. It is of interest to note that some compounds displayed a higher potency in the [^125^I]RHM-4 assay than the [^3^H]DTG binding assay, which supports the conclusions of Abbas et al. [24] regarding the limitations of [^3^H]DTG in screening ligands for affinity for the σ2R/TMEM97.

To explore the large difference in the σ2R/TMEM97 binding affinity between [^3^H]DTG and [^125^I]RHM-4, the cold RHM-4 derivative, ISO-1, was fragmented into two smaller substructures (**1** and **2**) and subjected to competition binding assays. Interestingly, **2** acts as the σ2R/TMEM97 binding fragment since its affinity is close to that of DTG, whereas **1** seems to play an important role in improving both the σ2R/TMEM97 binding affinity and selectivity versus σ1R. To further explore the influence of the secondary binding site on σ2R/TMEM97 affinity, two derivatives from RHM-4 were synthesized and screened in sigma receptor binding assays. Both **3** and **4** showed very high affinity for σ2R/TMEM97 and good selectivity versus σ1R, indicating the importance of the secondary binding site in developing high-affinity σ2R-specific compounds.

In summary, σ2R/TMEM97 is an important protein that has been linked to cancer and a number of neurological disorders. It has also been shown to play a key role in the internalization of LDL and other lipoproteins by forming a trimeric complex with PGRMC1 and LDL receptor or LRP. Since [^125^I]RHM-4 has a high binding affinity and high selectivity for σ2R/TMEM97 versus σ1R, it should be useful in probing the function of this protein using in vitro binding assays. The results of this study also indicate that [^125^I]RHM-4 is an improved ligand for use in radioligand binding studies for screening new σ2R/TMEM97 ligands. Finally, our results support the presence of two different binding sites in the σ2R/TMEM97, the “DTG binding site”, which is important for binding to the σ2R/TMEM97, and the secondary binding site, which is important for generating ligands having a high affinity and selectivity for σ2R/TMEM97 versus σ1R.

## 4. Materials and Methods

### 4.1. Reagents

All chemicals and solvents were purchased from Sigma-Aldrich (St. Louis, MO, USA). RHM-4, SV119, and **2** were synthesized according to previously published methods [25,26,27,28,29]. The synthesis of **1**, **3,** and **4** are provided in the Supplementary Material [30,31]. [^3^H]DTG, with a specific activity of 39 Ci/mmol, was purchased from Perkin Elmer (Boston, MA, USA). [^125^I]RHM-4 was radiolabeled according to a previously published method [32].

### 4.2. Liver Membrane Preparation

Liver membranes were prepared as previously described with minor modifications [33]. Briefly, the livers of female SD rats (Rockland, ME, USA) were dissected and homogenized using a Wheaton overhead stirrer (120 Vac Overhead Stirrer, Millville, NJ, USA) in 10 mL/g tissue weight of ice-cold 10mM Tris-HCl/0.32M Sucrose, pH 7.4. The crude homogenate was centrifuged for 10 min at 1000× *g* at 4 °C, the pellets were discarded, and the supernatant was further centrifuged at 31,000× *g* for 20 min at 4 °C. The supernatant was discarded, and the pellet was resuspended in 3 mL/g of ice-cold 10 mM Tris-HCl/0.32M sucrose, pH 7.4, and centrifuged again at 31,000× *g* for 20 min at 4 °C. After centrifugation, the pellets were resuspended in 1 mL of 50mM Tris-HCl, pH 7.4, and stored at −80 °C.

### 4.3. Saturation Binding Assays

The saturation binding protocols for [^3^H]DTG and [^125^I]RHM-4 have been previously described [4,6]. The SD rat liver membranes were incubated with [^3^H]DTG (0.5–130 nM) for 120 min or with [^125^I]RHM-4 (0.02–9 nM) for 90 min at room temperature. Nonspecific binding in both studies was defined as the presence of 10 μM DTG; 100 nM of (+)-pentazocine was added to the [^3^H]DTG binding study to mask the sigma-1 binding site. After incubation, the bound ligands from both studies were filtered using an M-24 Brandel filtration system (Brandel, Gaithersburg, MD, USA), collected on glass fiber papers (Whatman grade 934-AH, GE Healthcare Bio-Sciences, Pittsburgh, PA, USA), and counted using a MicroBeta2 Microplate counter 2450 or Wizard2 Automatic Gamma Counter 2470. All Kd and Bmax values were calculated via a nonlinear regression method using Prism, and the protein concentrations were determined using Lowry et al.’s method [34] with BSA as the standard.

### 4.4. Competitive Receptor Binding Assays

The protocols for the [^3^H]DTG (Perkin Elmer, Boston, MA, USA) and [^125^I]RHM-4 binding assays were performed as previously described [13,32]. Briefly, 30–60 μg of the liver membrane from male SD rats and different concentrations of the nonradioactive ligands (1 μM to 1 nM) were incubated with [^3^H]DTG (5 nM) or [^125^I]RHM-4 (0.1 nM) for 120 min or 90 min at room temperature, respectively. The nonspecific binding was defined with 10 μM DTG (Sigma-Aldrich, St. Louis, MO, USA); 100 nM of (+)-pentazocine (Sigma-Aldrich, St. Louis, MO, USA) was added for [^3^H]DTG binding to mask the σ1R receptor. After incubation, the bound ligands were filtered using an M-24 Brandel filtration system (Brandel, Gaithersburg, MD, USA), collected on glass fiber paper (Whatman grade 934-AH, GE Healthcare Bio-Sciences, Pittsburgh, PA, USA), and counted using a MicroBeta2 Microplate counter 2450 (Perkin Elmer, Boston, MA, USA) or Wizard2 Automatic Gamma Counter 2470 (Perkin Elmer, Boston, MA, USA). The inhibition constant [14] values of the tested ligands were determined using the Cheng and Prussoff equation [35], and the mean Ki values ± SEM were reported for at least three independent experiments.

For the σ1R assay, [^3^H]Pentazocine (Perkin Elmer, Boston, MA, USA) was used as the radioligand to define the σ1R binding affinity of the test compounds, and the protocol followed the references published before with minor modification [36,37]. Briefly, 100 μg of the brain membrane from male Guinea pigs and the different concentrations of the nonradioactive ligands (1 μM to 1 nM) were incubated with [^3^H]Pentazocine (5 nM) for 90 min at 37 °C. The nonspecific binding was defined with 10 μM Haloperidol (Sigma-Aldrich, St. Louis, MO, USA). Afterward, the filter paper collection, signal counting procedure, and data analysis were in line with the study of [^3^H]DTG mentioned above.

## 5. Conclusions

Based on the data presented herein, [^125^I]RHM-4 is expected to provide a more stringent screening procedure for new σ2R/TMEM97 ligands compared to the nonselective radioligand [^3^H]DTG. Our present data suggest that the secondary binding pocket should be taken into consideration when designing new σ2R/TMEM97 compounds in the future to increase both the affinity and selectivity for σ2R/TMEM97 versus σ1R.

## Figures and Tables

**Table 1:** Different inhibition constants (Ki, nM) of the nonradioactive ligands competing with [^3^H]DTG or [^125^I]RHM-4 using SD rat liver membrane.

Ligand	[^3^H]DTG	[^125^I]RHM-4
DTG	19.0 ± 4.7	25.1 ± 10.2
RHM-4	11.7 ± 2.4	0.2 ± 0.1
Haloperidol	20.7 ± 8.1	22.2 ± 13.6
PB-28	1.1 ± 0.3	0.1 ± 0.0
SV119	44.3 ± 7.3	35.0 ± 4.0
Ifenprodil	5.3 ± 1.1	0.4 ± 0.2
AG-205	807.0 ± 237.6	570.6 ± 82.1
Elacridar	11.7 ± 0.7	0.9 ± 0.3
Ro 48-8071	369.8 ± 230.2	73.0 ± 32.7

Values are presented as means ± S.E.M.

## Data Availability

Data is contained within the article and supplementary material.

## Supplementary Materials

The following supporting information can be downloaded at: https://www.mdpi.com/article/10.3390/ph15121564/s1.
