# Supplementary material for: Characterization of Sigma-2 Receptor—Specific Binding Sites Using [3H]DTG and [125I]RHM-4
Source: Pharmaceuticals (Basel). 2022 Dec 15;15(12):1564. doi: 10.3390/ph15121564 (PMC9784403; doi:10.3390/ph15121564)
Supplement: Supplementary file 1 [file pharmaceuticals-15-01564-s001.zip › pharmaceuticals-2050125-supplementary.pdf]

Characterization of Sigma-2 receptor-specific binding identities using [^{3}H]DTG and [^{125}I]RHM-4

Weng CC[1,2,3], Riad A[3], Lieberman BP[3], Xu K[3], Peng X[3], Mikitsh JL[3], and Mach RH[3,*]

Supplementary Material

N-Butyl-2-(2-fluoroethoxy)-5-methylbenzamide (1)

2-(2-Fluoroethoxy)-5-methylbenzoic acid[1] (39.6 mg; 0.2 mmol) and O-(1H-6-Chlorobenzotriazole-1-yl)-1,1,3,3-tetramethyluronium hexafluorophosphate (103 mg; 0.25 mmol) were dissolved in N,N-dimethylformamide (2 mL). N,N-diisopropylethylamine (0.2 mL) was added. The mixture was kept stirring at 0 °C for 10 min. A solution of n-butylamine (30 µl, 0.3 mmol) in N,N-dimethylformamide (1 mL) and N,N-dimethylformamide (0.2 mL) was added at 0 °C and the mixture was stirred at room temperature overnight. The mixture was diluted with ethyl acetate (20 mL) and washed with H_2O (10 mL), $NaHCO_3$ aqueous (10 mL x 2) and brine (10 mL x 2). The organic layer was dried over Na_2SO_4 and concentrated under reduced procedure. The residue was purified by flash chromatography (CH_2Cl_2/MeOH 0-10%) yielding **1** as colorless solid (35.5 mg; 70%). ^{1}H NMR (500 MHz, $CDCl_3$): δ 8.01 (s, 1H), 7.88 (br s, 1H), 7.21 (dd, *J* = 8.5, 2.5 Hz, 1H), 6.83 (d, *J* = 8.5 Hz, 1H), 4.74-4.85 (m, 2H), 4.27-4.35 (m, 2H), 3.44-3.48 (m, 2H), 2.33 (s, 3H), 1.56-1.63 (m, 2H), 1.40-1.44 (m, 2H), 0.95 (t, *J* = 7.5 Hz, 3H). MS (ESI) *m/z* 254.3 $(M+H)^+$.

2-(2-Fluoroethoxy)-5-methylbenzaldehyde oxime

2-(2-Fluoroethoxy)-5-methylbenzaldehyde[1] (0.9 g; 5 mmol) was dissolved in 1:1 ethanol/water (50 mL), then hydroxylamine hydrochloride (416 mg; 6 mmol) and NaOH (240 mg; 6 mmol) were added. After the mixture was stirred for 10 h, excess ethanol was removed under reduced pressure. The residue was extracted by CH_2Cl_2 (30 mL × 3). The organic solution was washed

with brine (30 mL), dried over Na_2SO_4 and concentrated under reduced pressure. The residue was purified on silica gel column chromatography using CH_2Cl_2/hexane (v/v) as the mobile phase to get the product as a yellow solid (780 mg; 80%), Mp: 101-103 °C. ^{1}H NMR ($CDCl_3$/300MHz) δ 9.55 (bs 1H), 8.54 (s 1H), 7.54 (s 1H), 7.11 (d *J*=8.4 Hz, 1H), 6.79 (d *J*=8.4 Hz, 1H), 4.75-4.78 (m 1H), 4.63-4.66 (m 1H), 4.23-4.26 (m 1H), 4.14-4.17 (m 1H).

2-(2-Fluoroethoxy)-N-hydroxy-5-methylbenzimidoyl chloride

2-(2-Fluoroethoxy)-5-methylbenzaldehyde oxime (740 mg; 3.75 mmol) was dissolved in N,N-dimethylformamide (10 mL), and N-chlorosuccinimide (500 mg; 3.75 mmol) was added carefully with stirring. After the mixture was stirred overnight at ambient temperature, 40 mL ethyl acetate was added to the flask. The organic solution was washed with water (10 mL × 4) and brine. The washed organic solution was concentrated under reduced pressure. The crude compound (quantitative yield) was obtained as a yellow oil. ^{1}H NMR ($CDCl_3$/300MHz) δ 10.23 (bs 1H), 7.50 (s 1H), 7.07 (d *J*=8.4 Hz, 1H), 6.75 (d *J*=8.4 Hz, 1H), 4.75-4.78 (m 1H), 4.63-4.66 (m 1H), 4.23-4.26 (m 1H), 4.14-4.17 (m 1H).

2-(5-Hexynyl)-6,7-dimethoxy-1,2,3,4-tetrahydroisoquinoline

This intermediate was synthesized via N-alkylation of 6,7-Dimethoxy-1,2,3,4-tetrahydroisoquinoline with Hexyn-5-yl 4-methylbenzenesulfonate as described above to obtain the product as yellow solid (61%). ^{1}H NMR ($CDCl_3$/300MHz) δ6.59 (s 1H), 6.52(s 1H), 3.84 (s 3H),3.83(s 3H), 3.55 (s 2H), 2.82 (t *J*=5.4 Hz, 2H), 2.70 (t *J*=6.3 Hz, 2H), 2.52 (t *J*=7.5 Hz, 2H), 2.50 (td *J*=7.2 Hz, *J*=2.7 Hz, 2H), 1.96 (t *J*=2.7 Hz, 1H), 1.72-1.67 (m 2H), 1.63-1.58 (m 2H).

5-(4-(6,7-Dimethoxy-3,4-dihydroisoquinolin-2(1H)-yl)butyl)-3-(2-(2-fluoroethoxy)-5-methylphenyl)-isoxazole (3)

2-(2-Fluoroethoxy)-N-hydroxy-5-methylbenzimidoyl chloride (324 mg; 1.4 mmol) and 2-(5-Hexynyl)-6,7-dimethoxy-1,2,3,4-tetrahydroisoquinoline (106 mg; 0.39 mmol) were dissolved in CH_2Cl_2 (20 mL) and triethylamine (2 mL). After the mixture was stirred overnight at ambient temperature, it was diluted with CH_2Cl_2 (40 mL) and washed with water (20 mL X 3) to remove triethylamine. The organic solution was then dried over Na_2SO_4 and concentrated under reduced pressure. The residue was purified by silica gel column chromatography using CH_2Cl_2/hexane/MeOH (100/100/1, v/v/v) as the mobile phase to afford **3** as a clear oil (82 mg; 45%). ^{1}H NMR ($CDCl_3$/300MHz) δδ7.75 (s 1H), 7.18 (d *J*=4.8 Hz, 1H), 6.86 (d *J*=6.3 Hz, 1H), 6.58 (s 2H), 6.52 (s 1H), 4.80-4.82 (m 1H), 4.68-4.70 (m 1H), 4.27-4.29 (m 1H), 4.20-4.22 (m 1H), 3.84 (s 3H), 3.82 (s 3H), 3.54 (s 2H), 2.80-2.87 (m 4H), 2.70 (t *J*=4.5 Hz, 2H), 2.55 (t *J*=5.4 Hz, 2H), 2.33 (s 3H), 1.80-1.85 (m 2H), 1.67-1.71(m 2H). Mp (oxalate salt): 128-130 ºC. Anal. ($C_{27}H_{33}FN_2O_4 \cdot H_2C_2O_4$) (Calculated: C: 62.35, H: 6.32, N: 5.01; Found: C: 62.11, H: 6.17, N: 5.05).

4-Fluoro-N-hydroxybenzimidoyl chloride

4-Fluorobenzaldehyde oxime[2] (650 mg; 3.75 mmol) was dissolved in N,N-dimethylformamide (10 mL), and N-chlorosuccinimide (500 mg; 3.75 mmol) was added carefully with stirring. After the mixture was stirred overnight at ambient temperature, 40 mL ethyl acetate was added to the flask. The organic solution was washed with water (10 mL × 4) and brine. The washed organic solution was concentrated under reduced pressure. The crude compound (quantitative yield) was obtained as a yellow oil. ^{1}H NMR ($CDCl_3$/300MHz) δ 8.63 (bs 1H), 7.81-7.86 (m 2H), 7.11 (t *J*=5.4 Hz, 2H).

5-(4-(6,7-Dimethoxy-3,4-dihydroisoquinolin-2(1H)-yl)butyl)-3-(4-fluorophenyl)isoxazole (4)

4-Fluoro-N-hydroxybenzimidoyl chloride (243 mg; 1.4 mmol) and 2-(5-Hexynyl)-6,7-dimethoxy-1,2,3,4-tetrahydroisoquinoline (106 mg; 0.39 mmol) were dissolved in CH_2Cl_2 (20 mL) and triethylamine (2 mL). After the mixture was stirred overnight at ambient temperature, it was diluted with CH_2Cl_2 (40 mL) and washed with water (20 mL X 3) to remove triethylamine. The organic

solution was then dried over Na_2SO_4 and concentrated under reduced pressure. The residue was purified by silica gel column chromatography using CH_2Cl_2/hexane/MeOH (100/100/1, v/v/v) as the mobile phase to afford **4** as a clear oil (91 mg; 57%). ^{1}H NMR ($CDCl_3$/300MHz) δ7.78-7.74 (m 2H), 7.13 (t, *J*=6.3 Hz, 2H), 6.59 (s 1H), 6.51 (s 1H), 6.27 (s 1H), 3.84 (s 3H), 3.82 (s 3H), 3.54 (s 2H), 2.81-2.87 (m 4H), 2.70 (t *J*=4.5 Hz, 2H), 2.55 (t *J*=5.1 Hz, 2H), 1.80-1.86 (m 2H), 1.68-1.74 (m 2H). Mp (oxalate salt): 190-192 ºC. Anal. ($C_{24}H_{27}FN_2O_3 \bullet H_2C_2O_4$) (Calculated: C: 62.39, H: 5.84, N: 5.60; Found: C: 62.43, H: 5.90, N: 5.54).

Reference

1. Tu, Z.; Li, S.; Cui, J.; Xu, J.; Taylor, M.; Ho, D.; Luedtke, R. R.; Mach, R. H. Synthesis and Pharmacological Evaluation of Fluorine-Containing D_3 Dopamine Receptor Ligands. *J. Med. Chem.* **2011**, *54*, 1555-1564.
2. Singh, I, Zarafshani, Z, Lutz, J-F, Heaney, F. Metal-Free "Click" Chemistry: Efficient Polymer Modification via 1,3-Dipolar Cycloaddition of Nitrile Oxides and Alkynes. Macromolecules 2009, *42*, 5411-5413.
